# Hepatocyte SLAMF3 reduced specifically the multidrugs resistance protein MRP-1 and increases HCC cells sensitization to anti-cancer drugs

**DOI:** 10.18632/oncotarget.8679

**Published:** 2016-04-11

**Authors:** Grégory Fouquet, Véronique Debuysscher, Hakim Ouled-Haddou, Mélanie Simoes Eugenio, Baptiste Demey, Amrathlal Rabbind Singh, Christèle Ossart, Mohammed Al Bagami, Jean-Marc Regimbeau, Eric Nguyen-Khac, Mickael Naassila, Ingrid Marcq, Hicham Bouhlal

**Affiliations:** ^1^ INSERM-ERi 24 (GRAP) Centre Universitaire de Recherche en Santé CURS, Université de Picardie Jules Verne, Cellulaire Centre Hospitalier Universitaire Sud, Amiens, France; ^2^ EA 4666 LNPC, Centre Universitaire de Recherche en Santé CURS, CAP-Santé (FED 4231) Cellulaire Centre Hospitalier Universitaire Sud, Amiens, France; ^3^ Service de Thérapie Cellulaire Centre Hospitalier Universitaire Sud, Amiens, France; ^4^ Service de Chirurgie Digestive Centre Hospitalier Universitaire Sud, Amiens, France; ^5^ Department of Microbiology, Dr. G. Venkataswamy Eye Research Institute, Aravind Medical Research Foundation, Madurai, India; ^6^ Service Hépato-Gastroenterologie, Centre Hospitalier Universitaire Sud, Amiens, France

**Keywords:** SLAMF3, drugs sensitization, MRP-1, HCC, multidrug resistance

## Abstract

Multidrug resistance MDR proteins (MRPs) are members of the C family of a group of proteins named ATP binding cassette (ABC) transporters. MRPs can transport drugs including anticancer drugs, nucleoside analogs, antimetabolites and tyrosine kinase inhibitors. Drugs used in HCC therapy, such as tyrosine kinase inhibitor sorafenib, are substrates of uptake and/or efflux transporters. Variable expression of MRPs at the plasma membrane of tumor cells may contribute to drug resistance and subsequent clinical response. Recently, we reported that the hepatocyte SLAMF3 expression (Signaling Lymphocytic Activation Molecule Family member 3) was reduced in tumor cells from hepatocellular carcinoma (HCC) compared to its high expression in adjacent tissues. In the present study, we make a strong correlation between induced SLAMF3 overexpression and the specific loss of MRP-1 expression and its functionalities as a drugs resistance transporter. No changes were observed on expression of ABCG2 and MDR. More importantly, we highlight a strong inverse correlation between MRP-1 and SLAMF3 expression in patients with HCC. We propose that the SLAMF3 overexpression in cancerous cells could represent a potential therapeutic strategy to improve the drugs sensibility of resistant cells and thus control the therapeutic failure in HCC patients.

## INTRODUCTION

Hepatocellular carcinoma (HCC) is the third leading cause of cancer deaths worldwide. Highest incidence is observed in East Asia and sub-Saharan region, despite the recent improvement in early detection by imaging techniques [1]. HCC has recently been treated by a new therapeutic approach using small molecules such as Sorafenib, which inhibits tyrosine kinases [2]. The main cause of HCC is chronic cirrhosis, which is often associated with an infection with hepatitis B virus or hepatitis C virus. Alcohol consumption or other liver metabolic diseases such as nonalcoholic fatty liver or hepatitis exposure to aflatoxins are also considered as etiological factors. Regardless of the etiology, only patients with HCC at an early stage can benefit from therapies such as radical surgical resection, liver transplantation, or the local percutaneous ablation. Therapy based on anti-multikinases such as, Sorafenib, is indicated only in HCC patients with residual liver function and preserved physical condition [3, 4]. Unfortunately, during the course of treatment, cancer cells develop resistance towards functionally and structurally different anticancer drugs by either acquired (due to host factors) or intrinsic (due to genetic or epigenetic) mechanisms [5, 6]. Use of Sorafenib as a reference treatment improves only slightly overall survival in patients estimated at 3 months. Limited therapeutic effect of Sorafenib could be due to the resistance of cancerous cells to drugs and to the adverse reactions of this molecule. Resistance to drug therapy remains a major challenge in HCC treatment despite successful advances using targeted therapies [3, 5]. Indeed, the effectiveness of chemotherapy for HCC is affected by the multidrug resistance (MDR) phenotype [5]. One of the mechanisms of resistance to molecules of chemotherapy is due to decreased absorption of these molecules and their increased efflux via membrane transporters proteins [7].

Several types of transporters are physiologically expressed in different cell types such as human hepatocytes, which take up endogenous substances and drugs across the sinusoidal membrane and release them in the bile [8, 9]. Several articles have reported the implication of organic cation transporter (OCT) 1 (encoded by the *SLC22A1* gene), OCT3 (*SLC22A3*) as well as the ATP-binding cassette (ABC) efflux transporters MDR1/P-glycoprotein (*ABCB1*), multidrug resistance protein (MRP, ABCC2), and breast cancer resistance protein (BCRP, ABCG2) in resistance to drugs which are commonly used in the HCC treatment. These transporters also confer resistance to anthracyclines, platinum drugs and sorafenib [7, 9-11]. Several studies have examined the expression of BCRP, MDR1, ABC drug efflux and OCT drug uptake transporter in the acquisition of drug resistance in HCC [7, 12-15]. MRP-1 expression in HCC was found to be associated with more aggressive tumor phenotype [14, 16]. Recently, Huang et al. reported that co-treatment with a BCRP/ABCG2 inhibitor greatly increased the cytotoxicity of sorafenib in HCC cells [17]. Taken together, decreased expression of efflux transporters would favor the accumulation of cytostatic drugs within the tumor cells and improve the drug efficiency. Thus patient-specific expression of uptake and efflux drug transporters may contribute to the optimization of the selection of HCC drugs and/or adjustment of dosing.

SLAMF3 belongs to signaling lymphocytic activation molecule family receptors (SLAMF-Rs) that trigger both inhibitory and activation signals in immune cells [18]. We have earlier reported the expression of SLAMF3 in hepatocytes and made a link between SLAMF3 high expression in HCC cells and low proliferation index and cell cycle arrest at G2/M [19]. SLAMF3 expression also inhibits ERK1/2, JNK and mTOR pathways and controls tumor progression of HCC-xenografts in animal model [19]. In addition, we have also reported that SLAMF3 controls cell cycle progression in a RB/PLK-1 dependent manner [20]. The identification of SLAMF3 and its potential role in HCC cells proliferation control prompted us to investigate other potential pathways involved in its anti-proliferative effect.

In the present study, we have evaluated the effect of SLAMF3 expression on MDR expression levels in HCC cells. Forced expression of SLAMF3 in HCC specifically and significantly decreased the expression of MRP-1 in HCC cells. Rhodamine 123 accumulation in SLAMF3 overexpressing cells and use of MRP-1 specific inhibitor MK-571 identified the effect of SLAMF3 on MRP-1 expression and function. The MRP-1 function was inhibited as proved by efflux MPR-1 testing activity using Rhodamine 123 accumulation test and by specific inhibition tests using MK-571 as MRP-specific inhibitor. Taken together, inducing over expression of SLAMF3 in cancerous hepatocytes could improve its sensitivity to drugs and could be a potent adjuvant to therapeutic strategy in HCC.

## RESULTS

### Cells from hepatocarcinoma express MDR transporters and SLAMF3 inhibits specifically MRP-1 expression

**Figure 1:**
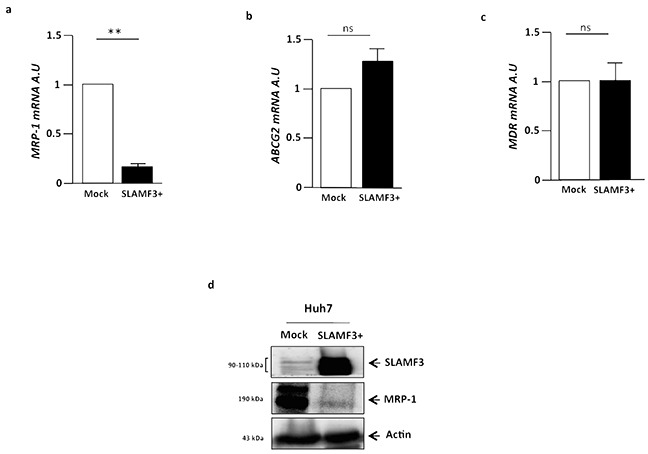
Cells from hepatocarcinoma express MDR transporters and SLAMF3 inhibited specifically MRP-1 expression Total mRNA from human HCC Huh-7 cells WT, free plasmid transfected cells (Mock) and SLAMF3 overexpressing cells transfected with SLAMF3 plasmid were extracted and MRP-1 **a.** ABCG2 **b.** and MDR **c.** genes expression was quantified by qRT-PCR. The results are presented as the mean ± SD from three independent experiments (n = 3; **p<0.01). **d.** Proteins extracted from mock and SLAMF3^+^ were analyzed by Western blot for MRP-1 expression (190 kDa). The SLAMF3 (90-110 kDa) expression level was shown for each condition mock and SLAMF3+ cells. One representative result from three independent experiments is shown.

Several studies have examined the expression of BCRP, MDR1, ABC drug efflux or OCT drug uptake transporter in the acquisition of drug resistance in HCC. Recently, Huang et al. reported that co-treatment with a BCRP/ABCG2 inhibitor greatly increased the cytotoxicity of Sorafenib in HCC cells [17]. We checked the expression of major drugs transporters implicated in the acquisition of drug resistance profile. Then, transcripts from human HCC cell line Huh-7 cells (untreated cells WT, Mock plasmid transfected and SLAMF3 overexpressing cells) were extracted and MDR, ABCG2 and MRP-1 genes expression was quantified by Q-PCR. We show that MDR, ABCG2 and MRP were highly expressed by Huh-7 cells (Figure 1a, 1b, 1c). We have earlier described the expression of SLAMF3 receptor in hepatocytes and shown that the high level expression of SLAMF3 inhibits proliferation in HCC cells [19, 20]. In order to clarify the link between the tumor suppressor effect of SLAMF3 and MDR expression in hepatocytes, we forced the SLAMF3 expression in Huh-7 cells and then quantified their expression. Interestingly among the three genes tested, only MRP-1 is specifically and significantly (*p<0.01*) decreased in hepatocytes when SLAMF3 was highly expressed and no effect was observed on MDR and ABCG2 expression level (Figure 1a, 1b, 1c). Observing the inhibition of MRP-1 mRNA expression, prompted us to quantify the protein levels. Lysates of cells transfected with mock and SLAMF3 plasmid were analyzed by Western blot. Cells transfected with SLAMF3 plasmid displayed significantly higher levels of SLAMF3 protein than mock transfected cells. However, in the cells transfected with the mock plasmid displayed elevated levels of MRP-1 protein than the SLAMF3 over expressing cells. (Figure 1d).

### MRP-1 is highly expressed in tumor tissues from HCC patients

**Figure 2:**
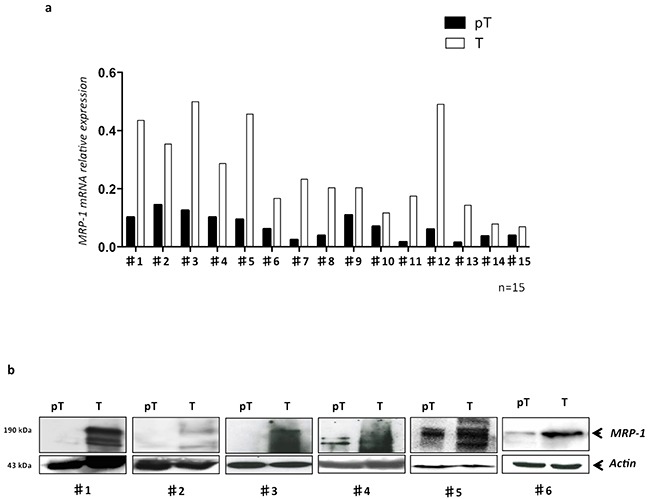
The MRP-1 is highly expressed in tumor tissues of HCC patients Total mRNA and proteins were extracted from freshly resected samples obtained from HCC patients (n=15) and MRP-1 expression was evaluated by qRT-PCR and WB. **a.** The MRP-1 mRNA level in each patient was shown; **b.** the MRP-1 protein (190 kDa) was detected by Western blot using specific anti-MRP-1 antibody in primary HCC samples in tumor (T) and peri-tumor (pT) areas from 6 patients with HCC.

Based on our *in vitro* results, we then quantified the MRP-1 in paired T (tumor) and pT (peri tumor) samples from 15 HCC patients freshly resected at Amiens University Hospital, France. Analysis of mRNA in samples demonstrated that in all patients (100%), the MRP-1 expression was significantly (*p<0.01*) higher in tumor tissues compared to pT (Figure 2a). The protein expression analysis by Western blot confirmed the higher expression of MRP-1 in T when compared to pT tissues (Figure 2b).

### Inverse correlation between SLAMF3 and MRP-1 expression in HCC patient samples and HCC cell lines

**Figure 3:**
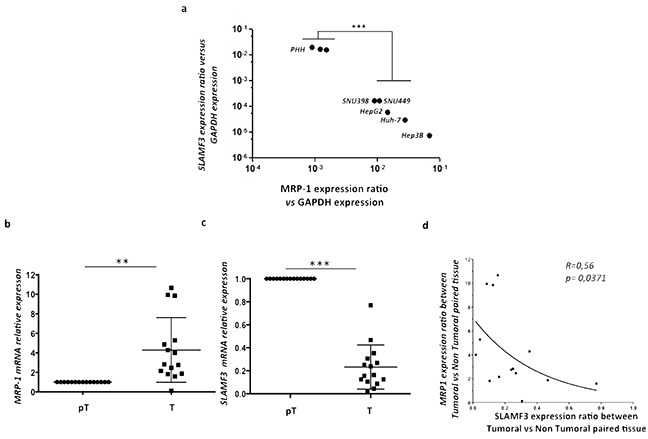
Inverse correlation between SLAMF3 and MRP-1 expression in HCC cell lines and primary hepatocytes Total mRNA from primary healthy hepatocytes PHH and different hepatocyte cancerous cell lines (Huh-7, HepG2, Hep3B, SNU398 and SNU449) was extracted and SLAMF3 and MRP-1 mRNA expression was quantified by qRT-PCR **a.** Transcripts were standardized by GAPDH quantification used as control. Results were presented as the mean of six independent experiments ± SD (n=6, ***p<0.001); **b, c.** MRP-1 and SLAMF3 mRNA, respectively, expression were analyzed in same extract from resected HCC patients by qRT-PCR. The mRNA quantities were compared between tumor (T) and peri-tumor (pT) areas and results presented as median, *p<0.05 for MRP-1 and ***p<0.005 for SLAMF3. The correlation between SLAMF3 and MRP-1 expressions was evaluated and represented as curve **d.** R= 0.56 and * p<0.05.

Expression of SLAMF3 is lost in tumor tissues as well as in cells from HCC cell lines compared to healthy tissues and cultured primary human hepatocytes PHH as we have reported previously [19, 20]. Here, we analyzed the expression of SLAMF3 and MRP-1 in cells from several HCC cell lines namely Huh-7, HepG2, Hep3B, SNU398, SNU449 and compared to PHH. We observed a strong inverse correlation between the expression of SLAMF3 and MRP-1 in PHH in which SLAMF3 is highly expressed while MRP-1 has weakly detectable. Inversely, in HCC cells, the expression of SLAMF3 is repressed with an elevated expression of MRP-1 (Figure 3a). Similarly, we also checked this correlation in pT/T primary tissue samples from HCC patients. The expression of SLAMF3 in all samples was checked and confirmed our previously reported results as SLAMF3 expression in T was reduced compared to pT tissues (Supplementary Figure S1). Our results confirm the significant (*R=0.56, p = 0.0371*) inverse correlation between SLAMF3 and MRP-1 expression in all patients with HCC (100%) (Figure 3b, 3c, 3d). This result suggested that the acquisition of resistance by cancerous cells correlates with progressive loss of SLAMF3.

### Overexpression of SLAMF3 in HCC cells induced specifically MRP-1 dysfunction

**Figure 4:**
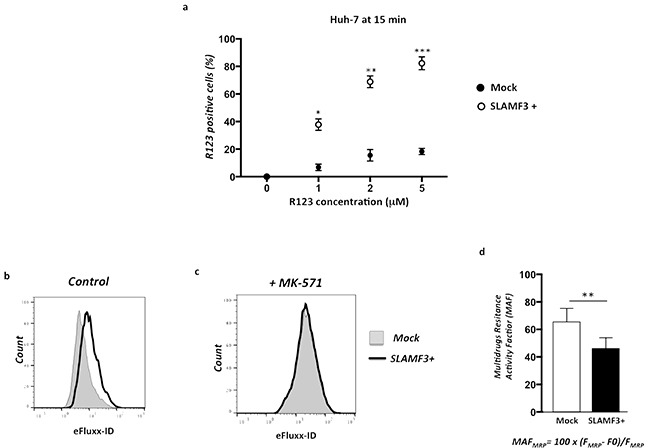
Overexpression of SLAMF3 in HCC cells blocks specifically MRP-1 function Cells transfected with SLAMF3 plasmid (SLAMF3+) and mock plasmid (2×10^6^/assay) were incubated with Rhodamine (R123) at 1, 2 and 5 mM for 15 minutes and R123 fluorescence measured in a flow cytometer by gating SLAMF3^+^ and mock. **a.** Results are presented as the mean ± SD from 3 independent experiments (n = 3; * *p< 0.05*, **p<0.01, ***p<0.005). The MRP-1 activity was specifically measured by using eFluxx-ID® Gold multidrug resistance assay kit (NZ-51030). Unless the eFluxx-ID® dye is pumped out of the cell, the esterase cleaved dye is trapped inside the cell. Cells exhibiting drug resistance will have diminished fluorescence. Cells (mock and SLAMF3+) were incubated with Gold detection reagent with and without specific inhibitor of MRP-1 (MK-571) for 30 min in 37°C and suspended in cold PBS for flow cytometry analysis. Fluorescence of gold dye(eFluxx-ID) is measured and presented as histogramsin mock (dotted line, full) and in SLAMF3 overexpressing cells SLAMF3+ (bold line, empty) in untreated cells **b.** and in the presence of MRP-1 specific inhibitor MK-571 **c.** One representative experiment from three (n=3) is presented. The formula of calculation of multidrug resistance activity factor (MAF) as: MAF_MRP_= 100 × (F_MRP_-F_0_)/F_MRP_ where F_MRP_ corresponds to the fluorescence intensity in the presence of MRP-1 specific inhibitor MK-571 and F_0_ to the fluorescence intensity in absence of inhibitor. Calculated MAF is presented **d.** as the mean ± SD from 3 independent experiments (n = 3; **p<0.01).

We have shown an inverse correlation between the SLAMF3 and MRP-1 expression in the liver tissue. The expression of MRP-1 in hepatic tissue confers to tumor a resistant profile to drugs whose discharge from the target cell is made by MRP-1. In addition, we highlight that SLAMF3 overexpression in the cancer cell induced, specifically, the downregulation of the MRP-1 expression. To clarify the effect on MRP-1 function, as drugs transporter, we checked the effect of SLAMF3 expression on Rhodamine 123 (R123) accumulation in Huh-7 cells (Mock and SLAMF3 overexpressing cells SLAMF3+). Briefly, R123 accumulates passively in the cell while its release from the cell is MDR-dependent [22]. Cells transfected with mock and SLAMF3 plasmid were incubated with R123 (1, 2 and 5 mM) for 15 minutes after several washes, cells were incubated in a R123 free medium for 2 hours at 37°C before cell recovery and analysis of R123 fluorescence in SLAMF3^+^ and mock sub-populations. We show that R123 accumulation levels in mock cells was significantly lower than that of SLAMF3^+^ (Figure 4a), suggesting that the MRP-1 downregulation induced by SLAMF3 overexpression prevents the efflux of the R123 that remains in the cancerous cells. eFluxx-ID® Gold multidrug resistance assay kit (ENZ-51030 Enzo Life Sciences (ELS) AG Lausen, Switzerland) used for simultaneous monitoring all for functional detection of all three relevant ABC transporter proteins namely, MDR (p-glycoprotein), MRP-1 and BCRP. Briefly, the assay uses a hydrophobic, non-fluorescent compound that readily penetrates the cell membrane, where it is hydrolyzed to a hydrophilic fluorescent dye by intracellular esterases. Unless the eFluxx-ID® dye is pumped out of the cell, the esterase cleaved dye remains trapped inside the cell. Thus, cells exhibiting drug resistance will exhibit a diminished fluorescence. The fluorescence signal of the dye generated within the cells thus depends upon the activity of the ABC transporters. The cells with highly active transporters exhibit lower fluorescence because of the active efflux of the probe from the cell. Application of specific inhibitors of the various ABC transporter proteins allows differentiation between the three common types of pumps. In our test, cells (mock and SLAMF3^+^) were incubated with Gold detection reagent with and without MRP-1 specific inhibitor (MK-571) according to the kit protocol for 30 minutes at 37°C and suspended in cold PBS for analysis by flow cytometry. The formula to calculate multidrug resistance activity factor (MAF) is described in the instruction manual of the MDR assay kit and also described previously [23]. We observed a low fluorescence in Mock cells while a significant shift was observed when cells overexpressed SLAMF3 (Figure 4b). The calculated multidrug resistance activity factor (MAF) indicated the significant (*p<0.01*) decreased MDR activity in SLAMF3 overexpressing cells. In the presence of MK-571, the accumulating fluorescence signal of the dye generated within the cells was similar in SLAMF3 overexpressing cells as well as in Mock indicating the MRP-1 inhibition specifically (Figure 4d, 4c).

## DISCUSSION

The MDR of tumors is one of the major obstacles leading to the failure of chemotherapy, refers to the fact that the cells can resist a variety of chemicals [5]. Understanding the MDR protein regulators in HCC is crucial to improve the effectiveness of chemotherapy. Physiologically, MRP-1 mediates the active efflux of a broad range of glucuronide, glutathione, and sulfate conjugates [24]. Based on its broad substrate range and ubiquitous tissue distribution, MRP-1 is now believed to be involved in many physiological and pathophysiological processes, including inflammatory responses and oxidative stress defense [25]. Physiological substrate of MRP-1 also includes cobalamin (vitamin B12), which was confirmed by vesicular transport experimentation and gene silencing studies [26]. In cancerous cells, MRP-1 can mediate resistance to a wide variety of anticancer drugs, including doxorubicin, methotrexate (MTX), vincristine, and etoposide and its expression level often indicates cancer aggressiveness [27-29]. Akimitsu et al. showed that aggressive breast carcinoma subtypes, display an overexpression of MRP-1 [30]. MRP-1 is highly expressed in non-small cell lung cancer (NSCLC) than in small cell lung cancer (SCLC) cell lines. Furthermore, overexpression of MRP-1 and MRP-3 is responsible for decreased drug sensitivity towards vincristine, etoposide, doxorubicin, and cisplatin in lung cancer patients [31]. The involvement of MRP-1 was also highlighted in colorectal carcinoma and level of MRP-1 was also found to be higher in patients with colorectal, prostate, pancreas and renal cancers as compared to the control group [32–35]. In the same sense, the expression of MRP-1 (mRNA and protein) was also associated with more aggressive tumor phenotype in hepatocarcinoma HCC [16]. In addition, clear correlation was also reported between higher MRP-1 expression levels and the poorly differentiated tumors as well as in large tumors showing microvascular invasion [36].

Recently, we identified the expression of SLAMF3 in hepatocytes and its implication in controlling proliferation of cancerous cell. We also highlighted certain potential mechanisms responsible for the tumor suppressor effect of SLAMF3 by inhibiting the MAPK/ ERK1/2 phosphorylation and blocking the cell cycle at G2/M in an RB/PLK-1 dependent-manner [19, 20]. Herein, we show that establishing the high expression of SLAMF3 in cancerous cells inhibits specifically the expression of MRP-1. In addition, we checked the cell sensitivity to Sorafenib in the presence of high and low expression of SLAMF3. Our results show that the IC50 of Sorafenib is respectively 4 and 5.4 μM in the presence and absence of SLAMF3 (data not shown). Our results suggest that Sorafenib is more efficient in the presence of a high expression of SLAMF3 through at least the inhibition of the expression and function of MRP-1. Our results were confirmed by the inverse correlation observed between the decreased SLAMF3 expression and increased MRP-1 expression in HCC patients. It has been reported that MRP-1 mRNA levels were higher in subclass A HCCs, which have a worse survival [36]. Taken together, our results suggest that the loss of SLAMF3 expression by cancerous cells may be one of the mechanisms by which transformed cells overexpress MDR, which result in drug resistance so that the cancer can escape chemotherapy.

On one hand, SLAMF3 inhibited MAPK/ ERK factors as we previously reported [19]. In our present work, we demonstrate the link between SLAMF3 expression and resistance to drugs. El Azreq et al. reported that MRP-1 expression was up-regulated in an MAPK/ ERK – dependent manner when β1-intergin signaling was triggered by collagen [37]. Our results suggest that one mechanism by which SLAMF3 may inhibit MRP-1 expression may implicate the inhibition of MAPK-ERK pathway. On the other hand, SLAMF3 induced the cell cycle blockade at S-G2/M stage by PLK-1 inhibition. We previously evidenced that RB as cell cycle suppressor, is involved in the SLAMF3–induced cell cycle arrest [20]. Elevated MRP-1 expression probably results in a growth advantage to cancerous cells by pumping toxic substances out of the tumor cells and protecting cells against oxidative stress-related damage. Combining chemotherapy with a MRP-1 inhibitor could improve the response of HCC to chemotherapy in cases with elevated MRP-1 expression. Here in, we show that SLAMF3 overexpression specifically reduced the MRP-1 function to pump drugs outside of cells. Therefore, developing inhibitors to block drug efflux function of MRPs could be a viable strategy especially for the patients selected with higher expression of MRPs.

Finally, our results suggest a potent role of SLAMF3 to increase sensitization of cancerous cells to drugs by inhibiting MDR protein MRP-1. The forced expression of SLAMF3 could be one of the potent therapeutic strategies to control tumor progression by controlling proliferation, induction of apoptosis and improving efficiency of drugs. The pathophysiological mechanisms that induce repression of SLAMF3 in tumorous cells still remain unknown and additional studies are needed to identify the molecular partners of hepatocyte SLAMF3 to elucidate mechanisms implicated in the tumor-suppressing functions.

## MATERIALS AND METHODS

### Patient samples, cell culture and treatments

Fifteen (n = 15) pairs of tumor (T) samples and matched peritumoral (pT) samples were obtained from HCC patients undergoing surgical resection at Amiens University Hospital (Amiens, France). Our protocol was approved by the local independent ethics committee (Comité de Protection des Personnes (CPP) Nord-Ouest, Amiens, France). Patients were provided with information on the study procedures and objectives and all gave their written consent to participation. The clinic and biological information are summarized in Table 1. Total mRNAs and proteins were extracted using specific kits and used to test SLAMF3 and MDR transporters expression.

**Table 1:** Clinical and biological parameters of HCC patients

Patients	Age	Sex	Tumor size (cm)	Cirrhosis (Y/N)	NASH (Y/N)	Virus (Y/N)	METAVIR Score
♯1	74	M	7×7×6	Y	Y	N	A1F4
♯2	59	M	5×5×5	Y	N	N	A1F4
♯3	63	M	16×8×8	Y	N	N	A2F4
♯4	63	M	13×12×10	N	N	N	A1F1
♯5	71	M	5.5×3×1	N	Y	N	A1F1
♯6	59	M	3.5×4.5×5	Y	Y	N	A1F4
♯7	65	M	24×18×15	N	N	N	A1F1
♯8	56	M	1.8×1.5×1.5	Y	Y	N	A1F4
♯9	61	M	5×4×4	N	Y	N	A1F2
♯10	59	M	9×5×5	N	N	Y	A0F0
♯11	NC	NC	NC	NC	NC	NC	NC
♯12	84	M	4×3×3	Y	N	N	A1F3
♯13	76	F	2×2×2	N	N	N	A2F4
♯14	NC	NC	NC	NC	NC	NC	NC
♯15	67	M	3×2×5	Y	N	N	A1F4

Age **Y**ears; Sex (M: **M**ale, F: Female); **NC** non-communicated; **Y**: Yes **N**: No; METAVIR Score **A**: Area, **F** Fibrosis.

Human HCC-derived cell line Huh-7 was obtained from virology department (Pr G. Duverlie, CURS, EA 4294 virology laboratory, CHU Sud, Amiens). Hepatocarcinoma cell lines HepG2, Hep3B, SNU 398 and SNU 449 were purchased from ATCC (Molsheim, France). Cells were maintained in DMEM (Life Technologies/Invitrogen, Saint Aubin, France) supplemented with 10% fetal calf serum (FCS) (PAA, Velizy-Villacoublay, France) and 1% penicillin/streptomycin (Life Technologies/Invitrogen, Saint Aubin, France). Primary Healthy Human Hepatocytes PHH (Lonza, Basel, Switzerland) cells were maintained in phenol red and serum-free HBCTM Basal Medium. HBCTM SingleQuots®Kit containing 500 μL hEGF, 500 μL transferrin, 500 μL hydrocortisone, 10 mL bovine serum albumin (BSA), 500 μL ascorbic acid, 500 μL GA-1000 and 500 μL insulin was added to basal medium (Lonza, Basel, Switzerland).

### Antibodies and reagents

For cytometry analysis, the Fluorescein isothiocyanate (FITC)- and Phycoerythrin (PE)-conjugated monoclonal anti-SLAMF3 (clone HLy9.1.25) and the matched isotype IgG1 were purchased from AbD Serotec (Colmar, France). For Western blot (WB) analysis, the anti-SLAMF3 (clone HLy9.1.25, AbD Serotec, Colmar, France) and mouse monoclonal antibody directed against human MRP-1 (clone MRPm5, Abcam, Paris, France), HRP-conjugated secondary antibodies were purchased from Cell Signalling Technology (Beverly, MA, USA). Anti-actin antibody (clone C-11) used as control for WB was purchased from Santa Cruz Biotechnology (Heidelberg, Germany).

### mRNA extraction, quantitative PCR, sequencing and plasmid construction

Total mRNA was extracted using RNeasy kit (Qiagen) and RT-PCR was performed using 100 ng of total RNA. Quantitative PCR was performed according to the Taqman Gene Expression protocol (Applied Biosystems) using the following primers for SLAMF3: For- TGGGACTAAGAGCCTCTGGAA-3′, Rev-ACAGAGATTGAGAACGTCATCTGG-3′ and MGB probe with 6-FAM (5′-CCCCAACAGTGGTGTC-3′). The transcription of GAPDH was measured as an endogenous housekeeping control. Hepatocyte SLAMF3 cloned into a pBud CE4.1 plasmid has been reported earlier [19]. For SLAMF3 over-expression, cells (0.3 × 10^6^) were first seeded into six-well plates 24 h prior to transfection. The cells were transfected with 0.8 μg of plasmid DNA using the FuGENE HD Transfection Reagent Kit (Roche, Meylan, France) according to the manufacturer's instructions. Cells were incubated for 48 h at 37°C before analysis of SLAMF3 expression by mRNA quantification, flow cytometry and WB. For quantitative reverse-transcription PCR (qRT-PCR), the following primers were used: MDR-1 For-TCATCCATGGGG CTGGACTT, Rev-CCTCCAGATTCATGAAGAACCC; ABCG2 For-GACTTATGTTCCACGGGCCT, Rev-TGCCACAGCAGTGGAATCTC; MRP-1 For-TTCTGGCTGGTAGCCCTAGT, Rev-ACAGGACAAGACGAGCTGAA; GAPDH: For-AAGGTGAAGGTCGGAGTCAA, Rev-CTTGACGGTGCCATGGAATT.

### Western blot analysis

Cells (10^6^ per assay) and primary tissues were lysed in Nonidet P40 (NP40) buffer (1% NP40, 50 mM Tris pH 7.5, 10% glycerol, 150 mM NaCL, 1 mM EDTA, 100 mM Na3VO6, 0.5 mM phenylmethanesulfonyl fluoride (PMSF), 5 mg/ml aprotinin, 5 mg/ml leupeptin and 2 mg/ml pepstatin) containing protease and phosphatase inhibitors (Roche). Equal amounts of each protein sample were separated by electrophoresis on SDS-PAGE, blotted onto nitrocellulose membranes (Bio-Rad, Munich, Germany) and blotted with antibodies against SLAMF3, MRP-1 and actin. Blots were developed with the enhanced chemiluminescence (ECL) system (Amersham Pharmacia Biotech).

### Rhodamin accumulation test and flow cytometry analysis

Rhodamine 123 (R123, Santa Cruz Biotechnology, Heidelberg, Germany) is a fluorescent dye that enters passively into cell reaching the mitochondria and its efflux is dependent on a wide variety of MDR [21]. Multidrug resistant cells demonstrate reduced accumulation of Rhodamine 123. Briefly, SLAMF3 transfected cells (at 24 hour post-transfection, 2×10^5^ cells/ assay) were incubated with R123 (1, 2 and 5 mM) compared to R123-untreated cells for 15 minutes at 37°C. After several washes in PBS to remove excess R123, cultured cells were replaced at 37°C for additional 2 hours. Then, cells were harvested in cold PBS/0.01% sodium azide/0.5% BSA, washed and incubated with fluorescent-conjugated antibody anti-SLAMF3 (clone HLy9.1.25, AbDSerotec, Colmar, France) for 30 min at 4°C. Cells were analyzed by FACSAria and FACSDiva software (BD Biosciences, Le Pont de Claix, France). Results were presented as R123 positive cells in SLAMF3^−^ and SLAMF3^+^ gated cells. R123 signal detected at 30 minutes in cells from cancerous lung cell line A549 was used as positive control.

### Direct dye efflux assay for multidrug resistance

For specific inhibition of MDR, we used eFluxx-ID® Gold multidrug resistance assay kit (NZ-51030) for simultaneous monitoring and functional detection of three relevant ABC transporter proteins: MDR1 (p-glycoprotein), MRP-1 and BCRP (ENZ-51030-K100, Enzo Life Sciences (ELS) AG Lausen, Switzerland). Briefly, 2×10^5^ cells (WT, mock and SLAMF3+) were incubated with Gold detection reagent with and without specific inhibitors of MDR1 (Verapamil), MRP-1 (MK-571) and BCRP (Noviobiocin) according to the protocol for 30 min in 37°C. Cells were suspended in cold PBS for flow cytometry analysis. The formula to calculate multidrug resistance activity factor (MAF) is described in the instruction manual of the MDR assay kit which is: MAF_MRP_= 100 × (F_MRP_-F_0_)/F_MRP_ where F_MRP_ corresponds to the fluorescence intensity in the presence of verapamil and F_0_ to the fluorescence intensity in the absence of inhibitor.

### Statistical analysis

Independent Student's t-test was used to compare MRP-1 mRNA expression in T and pT samples. The Mann-Whitney U test was used for correlations between (Spearman) between MRP-1/SLAMF3 mRNA expression. Unless otherwise stated, results are expressed as the mean ± SD. Statistical analyses (Mann-Whitney tests and an analysis of variance) were performed with Prism software (version 4.0, GraphPad Inc., San Diego, CA, USA). The threshold for statistical significant was set to p <0.05 for all analyses.

## SUPPLEMENTARY FIGURES


