# Supplementary material for: Hepatocyte SLAMF3 reduced specifically the multidrugs resistance protein MRP-1 and increases HCC cells sensitization to anti-cancer drugs
Source: Oncotarget. 2016 Apr 11;7(22):32493–503. doi: 10.18632/oncotarget.8679 (PMC5078028; doi:10.18632/oncotarget.8679)
Supplement: Supplementary file 1 [file oncotarget-07-32493-s001.pdf]

Hepatocyte SLAMF3 reduced specifically the multidrugs resistance protein MRP-1 and increases HCC cells sensitization to anti-cancer drugs

SUPPLEMENTARY FIGURE

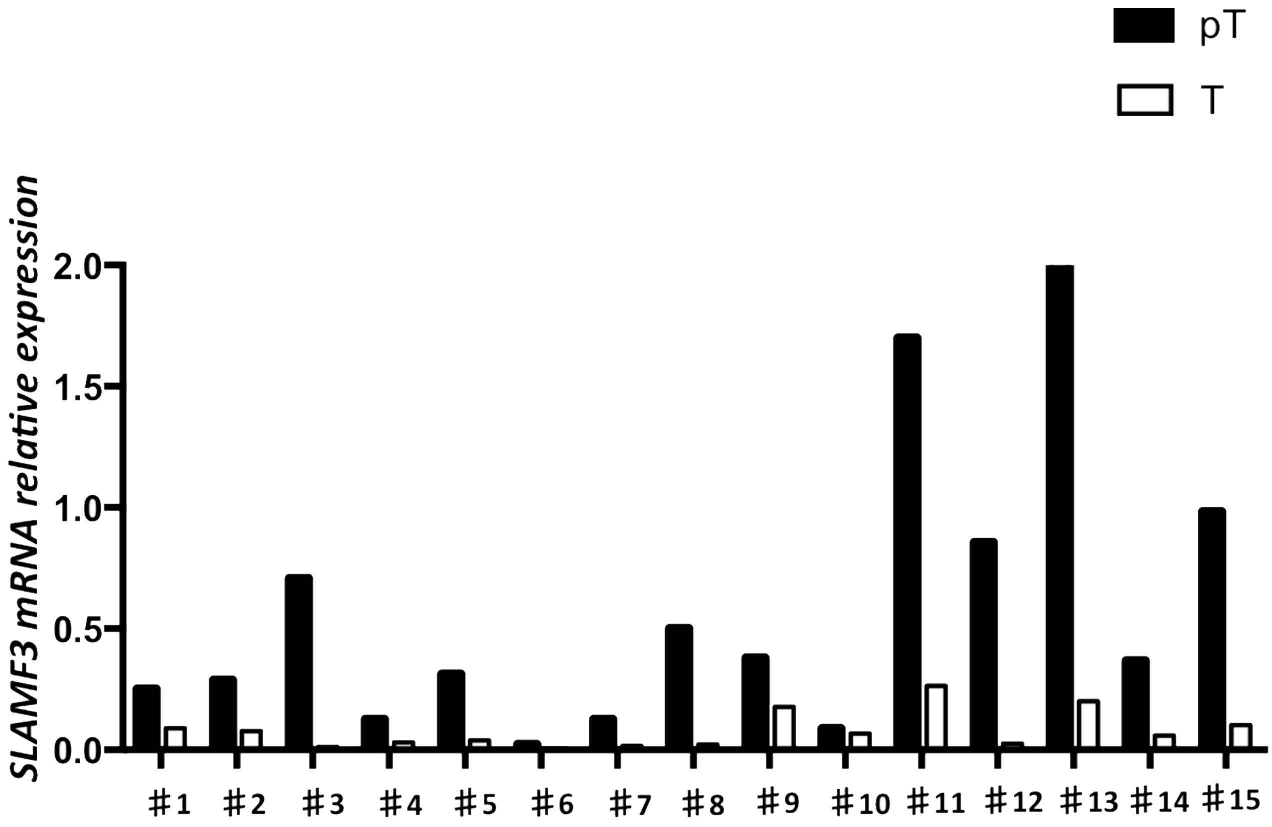

Supplementary Figure S1: Expression of SLAMF3 transcripts in patients with HCC. The mRNA quantification by qRT-PCR of SLAMF3 was carried out in 15 (n=15) of paired pT /T samples from HCC patients. Results are presented for each patient independently.
